# Parenting sense of competence and its predictors among primiparous women: a longitudinal study in China

**DOI:** 10.1186/s12884-022-04881-y

**Published:** 2022-07-07

**Authors:** Yi Zhu, Xuan Zhou, Xiaoxu Yin, Lei Qiu, Na Sun, Rongrong An, Yanhong Gong

**Affiliations:** 1 Department of Social Medicine and Health Management, School of Public Health, Tongji Medical College, Huazhong University of Science and Technology, Wuhan, P. R. China; 2 Department of Anesthesiology, Tongji Hospital, Tongji Medical College, Huazhong University of Science and Technology, Wuhan, P. R. China; 3 Department of Epidemiology, School of Public Health, Hainan Medical University, Haikou, P. R. China

**Keywords:** Primiparous women, Parenting sense of competence, Self-efficacy, Postpartum period, Postpartum care, China, Longitudinal study

## Abstract

**Background:**

Parenting sense of competence significantly affects the quality of parenting behaviours and healthy infant development. However, primiparous women without parenting experience may lack confidence and feel stress. This study aimed to explore the status of parenting sense of competence and identify its predictors among primiparous women.

**Methods:**

A longitudinal study design was used. Primiparous women were recruited by using a convenience sample from two women’s and children’s hospitals in two cities in China. All primiparous women completed questionnaires on demographic characteristics, infant characteristics, family function, and parenting sense of competence at 1 month postpartum. At 3 months postpartum, each women’s parenting sense of competence was re-assessed. Generalised linear regression was applied to identify the predictors of parenting sense of competence at 3 months postpartum.

**Results:**

A total of 743 Chinese primiparous women were included in the analysis. The average parenting sense of competence score of the participants at 3 months postpartum was 70.18 (SD = 12.33). According to the generalised linear regression analysis, higher levels of parenting sense of competence at 3 months postpartum were significantly associated with older age (β = 0.13, *P* = 0.005), better family function (β = 0.37, *P* <  0.001), and higher levels of parenting sense of competence (β = 0.35, *P* <  0.001) at 1 month postpartum. In contrast, lower levels of parenting sense of competence at 3 months postpartum were associated with poorer self-rated economic status (β = − 0.16, *P* = 0.027), poorer infant health (β = − 0.26, *P* = 0.007), and mixed or formula feeding (β = − 0.11, *P* = 0.018) at 1 month postpartum.

**Conclusions:**

Chinese primiparous women have a relatively good parenting sense of competence, but there is still room for improvement. Maternal age, economic status, family function, infant health, and feeding patterns were significant predictors. To improve their parenting sense of competence, more attention should be paid to primiparous women who are young, with poor economic status, having an unhealthy infant, and mixed or formula feeding. In addition, measures should also be taken during the early postpartum period to improve family function.

**Supplementary Information:**

The online version contains supplementary material available at 10.1186/s12884-022-04881-y.

## Background

Transitioning to motherhood is a challenging period following childbirth because mothers face not only the pressure from changes in physical, psychological, and social relations, but also complex parenting tasks [1–3]. The acquisition of parenting confidence and competence during this transition is considered an essential component of the adaptation to maternal role [4]. Parenting sense of competence is the perceived self-efficacy and satisfaction of mothers in their maternal role [5], reflecting the belief that they can perform the maternal role effectively [6]. Parenting sense of competence is not only a factor affecting maternal mental health [7], but also a strong predictor of parenting ability and infant outcomes [8]. It is difficult for mothers with lower levels of sense of competence in parenting to adapt to the maternal role or to overcome parenting stress. These mothers are more prone to negative emotions such as anxiety and depression, which affect maternal physical and mental recovery and the healthy development of infants [9, 10]. In contrast, mothers with higher levels of parenting sense of competence are able to complete the transition to motherhood more quickly, and they are more likely to adopt positive strategies to address challenging parenting tasks, such as toilet-training, caring for a crying or sick infant, and remediating infant’s behavioural problems, which can lead to improved maternal and infant health and well-being [11, 12]. Thus, it is necessary to understand the levels of parenting sense of competence and its associated factors*.*

In the early postpartum period, it is a great responsibility and burden for primiparous women without parenting experience to take on childcare tasks [13, 14]. Identifying the predictors of their parenting sense of competence is a prerequisite for formulating intervention strategies to improve parenting abilities. Several studies carried out worldwide have found that maternal demographic characteristics, psychological factors, and infant factors contributed notably to parenting sense of competence [14–19]. However, the results of studies in different countries and regions have been inconsistent. For example, a cross-sectional study conducted in Finland showed that maternal age was not significantly associated with parenting sense of competence [20], whereas several cohort studies conducted in Sweden and the United States identified age as an important factor influencing parenting self-efficacy and satisfaction in the maternal role [21, 22]. Furthermore, some studies on the relationship between family function and parenting sense of competence also reported discrepant findings. An early study found that good family function can improve parenting self-efficacy [23]. However, a recent study argued that there was no significant relationship between them [24]. The disparity in the social and cultural determinants in different regions may account for these differences. Accordingly, further studies are required to explore the factors affecting parenting sense of competence in different regions.

China is the world’s most populous country, with approximately 8.87 million new-borns in 2021 [25]. Since the long-term implementation of the One-Child Policy, more than half of parturient women have been primiparas in China [26]. Previous studies have found that Chinese primiparous women faced greater parenting stress, especially difficulties in mother-infant interactions and infant care, which severely limited the development of maternal roles and behaviours [27, 28]. However, few studies have focused on Chinese primiparous women’s parenting sense of competence and its related factors. Most of the existing studies have been cross-sectional, which limits the examination of the longitudinal association between variables and is not conducive to clarifying intervention factors [29, 30]. Although a few studies have been longitudinal, it is difficult to accurately assess whether the variable of interest is an independent predictor of parenting sense of competence as these studies did not control for baseline levels of parenting sense of competence [31, 32]. More rigorous researches are necessary to identify predictors of parenting sense of competence to develop targeted intervention strategies. A previous study found that the level of parenting sense of competence would stabilise around 3 months postpartum. During this period, the parenting sense of competence and satisfaction was more likely to be accurately measured [33]. Therefore, based on a prospective longitudinal study design, this study aimed to investigate the levels of parenting sense of competence at 3 months postpartum among Chinese primiparous women and to explore the predictors at 1 month postpartum related to parenting sense of competence at 3 months postpartum after controlling for baseline levels. Our results can provide a reference for maternal and child health workers to implement early postnatal targeted interventions. We hypothesised that demographic characteristics, family function, and infant characteristics in the early postpartum period would be predictors of parenting sense of competence at 3 months postpartum among primiparous women. This study not only helped to understand the levels of parenting sense of competence of Chinese primiparous women, but also provided representative data from China to understand the current state of parenting sense of competence globally.

## Methods

### Design

This study was part of a prospective longitudinal study conducted to explore the intention and behaviour of delivery modes among pregnant Chinese women [34]. The data were collected in the main study at seven time points: the first, second, and third trimesters of pregnancy and 1, 3, 6, and 12 months after childbirth. Considering the aim of our study, the data at 1 and 3 months after childbirth were used to identify the predictors of parenting sense of competence at 3 months postpartum among Chinese primiparous women.

### Procedure and participants

The prospective longitudinal study was conducted using a convenience sample from two public hospitals in two cities in China. The two public hospitals were Wuhan and Qianjiang women’s and children’s hospitals, respectively. The participants were recruited and followed between September 2018 and August 2020. During the recruitment period, the researchers invited women who arrived for routine maternity visits to participate in the study and complete baseline questionnaires. A follow-up questionnaire was sent to each woman via email or the social media platform WeChat. When participants did not have email accounts, we conducted the follow-up survey via the WeChat platform which is currently the most popular instant-messaging platform in China covering approximately 90% of the Chinese population [35]. Eligible participants were primiparous women, at least 18 years of age, and with singleton pregnancies. Participants were excluded if they were cognitively impaired or unable to complete the questionnaires. During the baseline survey, obstetricians first identified potential subjects based on the inclusion and exclusion criteria and recommended those who met the requirements to participate in our study. We contacted 1173 women referred by obstetricians, and 120 of them declined to participate in the survey. Finally, a total of 1053 women completed the survey during their first trimester. During the follow-up period, of the 1053 women, 931 completed the second-trimester follow-up assessment, 856 completed the third-trimester follow-up assessment, 794 completed the 1-month postpartum follow-up assessment, and 743 completed the 3-month postpartum follow-up assessment. According to the purpose of the study, 743 women who completed data collection at 1 month and 3 months postpartum were selected and included for analysis in this study. There were no differences in demographic characteristics between primiparous women who completed follow-up at 3 months postpartum and those who lost to follow-up (see Additional file 1).

As this study was part of a prospective longitudinal study, no sample size was calculated a priori specifically based on the topic of parenting sense of competence. To ensure that the sample size of this study was large enough to be able to perform valid statistical tests, we performed a post-hoc power analysis using G*Power (version 3.1.9.7) to estimate the statistical power of the samples based on the recommendation provided by Faul et al. [36] Our post-hoc analysis revealed that the statistical power for this study was all close to 1 for detecting a small (d = 0.2), a medium(d = 0.5), or a large effect size (d = 0.8) with a significance level of α = 0.05. Thus, the obtained sample size (*n* = 743) was large enough to detect linear regressions to assess the significant predictors of parenting sense of competence.

### Measures

Data were collected using structured, self-administered questionnaires. The 1-month postpartum questionnaire was developed based on a review of previous literature [19, 37] to investigate demographic characteristics, infant characteristics, maternal family function, and other factors that may influence the parenting sense of competence at 3 months postpartum. To control for the effect of baseline levels of parenting sense of competence on parenting sense of competence at 3 months postpartum, this study also investigated the levels of parenting sense of competence at 1 month postpartum. Parenting sense of competence was re-assessed at 3 months postpartum.

Demographic data on primiparous women included age, education level, employment status, attending antenatal education, self-rated sleep quality, self-rated economic status, and self-rated health status. Infant characteristics included type of delivery, infant gender, gestational weeks of infant, infant health, and feeding patterns.

Primiparous women’s family function was measured using the ‘Family Adaptability, Partnership, Growth, Affection, and Resolve (Family APGAR)’ index, which is a self-rated instrument designed by the American scholar Smilkstein [38]. It consists of five questions, each of which is scored on a 3-point Likert scale ranging from 0 (hardly ever) to 2 (often). The total score ranges from 0 to 10. A higher score indicates that a mother receives more family support. The scores of 7–10, 4–6, and 0–3 respectively represent good family function, moderate family dysfunction, and serious family dysfunction. The Family APGAR index, which has good reliability and validity, has been widely used in China [39]. In the current study, the Family APGAR index demonstrated a high internal consistency (Cronbach’s alpha **=** 0.83).

Primiparous women’s parenting sense of competence was assessed using the Parenting Sense of Competence scale. This scale, developed by Gibaud-Wallston, is a 17-item scale designed to measure maternal parenting self-esteem [40]. This scale consists of two subscales: the Efficacy subscale with eight items is designed to measure maternal perceptions of the knowledge and skills required for parenting, and the Satisfaction subscale with nine items is used to evaluate maternal satisfaction and comfort with their parenting role. Each question is answered using a 6-point Likert scale, ranging from ‘strongly disagree’ to ‘strongly agree’ coded with values from 1 to 6. The total score is the sum of the scores of all 17 questions and ranges from 17 to 102, with higher scores indicating higher competence in parenting. The scores of the Efficacy subscale and Satisfaction subscales range from 8 to 48 and 9 to 54, respectively. The Chinese version of the Parenting Sense of Competence scale has high internal consistency [41]. The Cronbach’s alpha of the Scale in this study was 0.89 at 1 month postpartum and 0.89 at 3 months postpartum, indicating good reliability.

### Statistical analysis

Statistical analysis was performed using the Statistical Analysis System (SAS) 9.4 for Windows (SAS Institute Inc., Cary, NC, USA). Descriptive analyses included means for continuous variables and percentages for categorical variables. T-test and analysis of variance were used to identify differences in parenting sense of competence scores at 3 months postpartum among groups with different maternal demographic characteristics, family function, and infant characteristics. A generalised linear regression model was used to examine the predictors of parenting sense of competence at 3 months postpartum among Chinese primiparous women after controlling for baseline levels of parenting sense of competence. All *P* values shown were two-tailed, with a significance level of 0.05.

## Results

Primiparous women’s demographic characteristics and their association with parenting sense of competence at 3 months postpartum are presented in Table 1. The mean age of the primiparous women was 27.20 years (range 18–41; SD **=** 3.17), and more than 70% had a college degree or higher. Almost two-thirds (64.20%) were employed, and less than two-fifths (19.92%) have attended antenatal education. The proportions of women who rated their sleep quality, economic status, and health status as good were 20.86%, 17.09%, and 38.22%, respectively. Additionally, approximately half of the women (49.93%) perceived good family function.

The mean score of parenting sense of competence at 3 months postpartum was 70.18 (range 30–102; SD **=** 12.33). Additionally, the mean score of self-efficacy was 33.92 (range 8–48; SD **=** 6.31), and the mean score of satisfaction was 36.26 (range 9–54; SD **=** 7.70). Univariate analysis showed that primiparous women who were older (*P* **=** 0.003), unemployed (*P* <  0.001), with better self-rated sleep quality (*P* <  0.001), with better self-rated economic status (*P* <  0.001), with better self-rated health status (*P* <  0.001), and with good family function (*P* <  0.001) had higher parenting sense of competence scores.

**Table 1:** Participants’ characteristics and their associations with parenting sense of competence at 3 months postpartum

Variables	All subjects		Parenting Sense of Competence at 3 months postpartum		***P***
	n	%	mean	SD	
**Total**	743	100	70.18	12.33	
**Age**					0.003
18–24	134	18.03	66.99	12.54	
25–30	516	69.45	70.71	11.88	
> 30	93	12.52	71.84	12.75	
**Education**					0.108
Junior high school or below	61	8.21	67.64	10.40	
Senior high school	108	14.54	68.62	10.53	
Junior college	527	70.93	70.63	12.86	
Master’s degree or higher	47	6.33	72.02	11.89	
**Employment status**					< 0.001
Employed	477	64.20	68.05	12.20	
Unemployed	266	35.80	71.37	12.25	
**Whether attended antenatal education**					0.220
Yes	148	19.92	70.46	12.35	
No	595	80.08	69.07	12.24	
**Self-rated sleep quality at 1 month postpartum**					< 0.001
Good	155	20.86	70.46	12.35	
Not good	588	79.14	69.07	12.24	
**Self-rated economic status at 1 month postpartum**					< 0.001
Good	127	17.09	73.97	14.13	
Not good	616	82.91	69.18	11.62	
**Self-rated health status at 1 month postpartum**					< 0.001
Good	284	38.22	76.09	13.35	
Not good	459	61.78	68.96	11.75	
**Family function at 1 month postpartum**					< 0.001
Good family function	371	49.93	72.69	12.81	
Serious family dysfunction	88	11.84	63.35	11.85	
Moderate family dysfunction	284	38.22	67.72	10.20	

Infant characteristics and their association with parenting sense of competence at 3 months postpartum are presented in Table 2. More than one-half (55.45%) of infants were born via normal vaginal delivery. The sex ratio was close to 1:1. 95.83% were term infants and 4.17% were premature infants. Most primiparous women (93.14%) perceived their infants’ health as good and the proportion of breastfeeding was 51.14%. Primiparous women who had a healthy infant (*P* < 0.001) and who breastfed (*P* = 0.043) had higher parenting sense of competence scores.

**Table 2:** Infant characteristics and their associations with parenting sense of competence at 3 months postpartum

Variables	All subjects		Parenting Sense of Competence at 3 months postpartum		***P***
	n	%	mean	SD	
**Type of delivery**					0.899
Normal vaginal delivery	412	55.45	70.23	12.62	
Cesarean section	331	44.55	70.12	11.97	
**Gender of infant**					0.510
Male	370	49.80	70.48	12.36	
Female	373	50.20	69.88	12.30	
**Gestational weeks of infant**					0.147
< 37	31	4.17	70.04	12.34	
37–42	712	95.83	73.32	11.82	
**Infants health status at 1 month postpartum**					< 0.001
Good	692	93.14	70.62	12.29	
Not good	51	6.86	64.25	11.35	
**Feeding patterns at 1 month postpartum**					0.043
Breastfeeding	380	51.14	71.08	12.58	
Mixed or formula feeding	363	48.86	69.25	12.01	

The results of the generalised linear regression analysis are presented in Table 3. Primiparous women’s parenting sense of competence at 3 months postpartum was predicted by maternal age, self-rated economic status, family function, infant health, feeding patterns, and parenting sense of competence at 1 month postpartum. Primiparous women who were older (β **=** 0.13, *P* **=** 0.005), with better family function (β **=** 0.37, *P* < 0.001) and better parenting sense of competence (β **=** 0.35, *P* < 0.001) at 1 month postpartum had higher levels of parenting sense of competence at 3 months postpartum. Primiparous women with poorer self-rated economic status (β **=** − 0.16, *P* **=** 0.027), with poorer infant health (β **=** − 0.26, *P* **=** 0.007), and mixed or formula feeding (β **=** − 0.11, *P* **=** 0.018) at 1 month postpartum had lower levels of parenting sense of competence at 3 months postpartum.

**Table 3:** Generalised linear regression analysis of predictors of parenting sense of competence at 3 months postpartum

Variables	Estimate	SE	t	***P***
**Constant**	2.68	0.29	86.56	<0.001
**Age**^**b**^	0.13	0.05	7.94	**0.005**
**Educational level (Ref** ^**a**^**: Junior high school or below)**				
Senior high school	0.04	0.10	0.18	0.669
Junior college	0.02	0.09	0.03	0.868
Master’s degree or higher	−0.06	0.13	0.23	0.635
**Employment status**^**b**^ **(Ref: Employed)**				
Unemployed	0.10	0.05	3.84	0.050
**Whether attended antenatal education (Ref: Yes)**				
No	−0.08	0.06	1.67	0.196
**Self-rated sleep quality at 1 month postpartum (Ref: Good)**				
Not good	−0.07	0.06	1.11	0.293
**Self-rated economic status at 1 month postpartum**^**b**^ **(Ref: Good)**				
Not good	−0.16	0.07	4.90	**0.027**
**Self-rated health status at 1 month postpartum (Ref: Good)**				
Not good	−0.10	0.05	3.61	0.058
**Family function at 1 month postpartum**^**b**^	0.37	0.05	63.19	**<0.001**
**Type of delivery (Ref: Normal vaginal delivery)**				
Cesarean section	−0.02	0.05	0.15	0.696
**Gender of infant (Ref: Male)**				
Female	−0.03	0.05	0.43	0.511
**Gestational weeks of infant (Ref:** < **37)**				
37–42	−0.07	0.12	0.31	0.576
**Infant health status at 1 month postpartum**^**b**^ **(Ref: Good)**				
Not good	−0.26	0.10	7.28	**0.007**
**Feeding patterns at 1 month postpartum**^**b**^ **(Ref: breastfeeding)**				
Mixed or formula feeding	−0.11	0.05	5.58	**0.018**
**Parenting sense of competence at 1 month postpartum**^**b**^	0.35	0.06	32.93	**<0.001**

^a^Ref is reference

^b^Variables significantly associated with parenting sense of competence

## Discussion

We assessed parenting sense of competence at 3 months postpartum among primiparous women and explored the predictors. Our findings suggested that maternal age, self-rated economic status, family function, infant health, feeding patterns, and parenting sense of competence at 1 month postpartum were predictors of parenting sense of competence at 3 months postpartum. This study used the Parenting Sense of Competence scale to evaluate Chinese primiparous women’s sense of competence and satisfaction in the maternal role. This scale has been employed to investigate the parenting competence of women in Hong Kong, China, with a Cronbach’s alpha of 0.87 [42]. The results of this study further indicated that the Parenting Sense of Competence scale has good reliability. Several studies carried out worldwide have used the same scale to assess parenting sense of competence. The mean scores reported by countries such as Nepal (64.34) [43] and Iran (58.72) [44] were considerably lower than that of this study (70.18), indicating that Chinese primiparous women’s sense of parenting competence was relatively good. However, room for improvement remains compared to Chinese multiparous women. A previous study from China found that the mean score of parenting sense of competence among Chinese multiparas was 72.70 [41], which is higher than the mean score for primiparous women reported in this study. Thus, appropriate intervention measures should be implemented to further improve the role competence and satisfaction of primiparous women.

The results of this study indicated that older primiparous women had higher levels of parenting sense of competence. Previous studies have also shown that maternal age was positively associated with parenting competence and satisfaction at 6 weeks postpartum [37] and parenting self-efficacy at 3 months postpartum [21]. Older primiparous women are more psychologically mature and have a wealth of life experiences and skills to cope with the developmental tasks of mothering. It is consistent with Bandura’s theory that mastering more experience contributed to the development of self-efficacy [45]. However, younger women are less likely to feel competent and satisfied in the maternal role [13]. These findings suggested that more attention should be paid to young primiparous women in postnatal care, providing them with personalised parenting support and care guidance to develop their maternal role competence and satisfaction.

According to our study, lower levels of parenting sense of competence were associated with poorer economic status. Economic income is not only an important source for primiparous women to obtain material support but is also an important guarantee for the use of parenting resources. Material support, such as paid leave and parenting subsidies, can improve maternal role competence by addressing the financial concerns of parenting and motivating mothers’ active participation in health education activities, as well as postnatal care services [20]. A study assessing the variation in parental happiness in 22 Organization for Economic Cooperation and Development (OECD) countries found that the financial demands in the parenting process can be effectively met and parenting satisfaction can be improved through the development policies for childcare-related welfare, such as subsidised parenting costs [46]. Thus, to alleviate the postnatal parenting burden among primiparous women, it is necessary to provide economic assistance for those in need.

This study also found that family function was an important factor affecting parenting sense of competence. Previous studies have shown that social support was effective in promoting maternal role change [47, 48]. Family support was a critical form of social support after discharge from hospital [12, 49]. Good family interactions, encouragement, and caring can relieve the stress and nervousness of primiparous women, thereby improving their sense of parenting competence. Singapore has implemented a family-centred nursing policy that supported family members to accompany and care for mothers during the perinatal period to facilitate their smooth transition to motherhood. This strategy has proven to be an effective way to improve maternal self-efficacy in self-care and new-born care [17]. Due to the significant impact of family function on the perception of competence in parenting, health personnel should develop intervention strategies that impact the whole family [19]. Family members such as husbands, mothers, and mothers-in-law of primiparous women should be encouraged to take an active part in infant care and share parenting knowledge or experience.

Moreover, primiparous women who perceived their infants had better health status and who breastfed may have higher levels of sense of parenting competence. Some studies have also confirmed that infant health was an important factor that affected parenting self-efficacy [23, 31]. Bandura’s theory stated that the perceived task difficulty was significantly related to self-efficacy [50]. If primiparous women perceived that their infants were in poor health and that it was difficult to complete parenting and caring tasks, their sense of parenting competence would be affected. Additionally, breastfeeding was associated with higher levels of parenting sense of competence. The possible reason for this finding was that breastfeeding increased the frequency of mother-infant interactions, which was considered to contribute to improving parenting sense of competence [51]. The frequency of mother-infant interactions may be reduced when mothers are formula or partial formula feeding which adversely affects parenting sense of competence. Some researchers have found evidence that early breastfeeding experiences were important predictors of increased maternal role competence and satisfaction [20, 52]. In an Internet-based intervention in Finland, maternal parenting behaviours and sleep quality were significantly improved by providing breastfeeding-related nursing guidance and health education, thus effectively improving their levels of sense of competence in parenting [53]. Unfortunately, our study found that the breastfeeding rate was only 51.14%, indicating that there is still some room for improvement in the breastfeeding rate among Chinese primiparous women. Therefore, interventions aimed at fostering maternal role competence and satisfaction should include efforts to encourage mothers to initiate and maintain breastfeeding. However, considering that some mothers were very stressed in maintaining breastfeeding or had difficulty continuing breastfeeding due to poor health [54], they should be encouraged to maintain positive mother-infant interactions through other means [51] to reduce the potential negative impact of breastfeeding cessation on parenting sense of competence.

Parenting sense of competence at 1 month postpartum was also an important predictor of parenting sense of competence at 3 months postpartum. This result further emphasised that early intervention is crucial. It was found that the levels of parenting sense of competence in the early postpartum period seemed unstable, which may be attributed to difficulties associated with coping with substantial physical and psychological changes and unfamiliar infant care tasks after childbirth [1, 33]. Around the third month postpartum, the levels of parenting sense of competence of primiparas stabilised after they had adequately experienced parenting tasks and maternal roles. Therefore, the first 3 months after childbirth may be a critical period to improve the sense of competence in parenting. Early postnatal interventions that target relevant factors will not only be effective in improving parenting sense of competence at 3 months postpartum, but also help to maintain stable long-term parenting self-efficacy and satisfaction. Further intervention studies are required to confirm the effectiveness of early postnatal interventions.

To the best of our knowledge, this study is the first to identify the predictors of Chinese primiparous women’s parenting sense of competence at 3 months postpartum using a prospective study design, which provides a scientific reference for improving their parenting ability and the quality of parenting behaviours. However, this study has several limitations. First, this study was conducted only in Hubei Province, China, which to some extent limits the representativeness of the study sample and the extrapolation of the study results. Thus, future research should examine whether these findings can be replicated across diverse regions and populations. Second, since the appropriate cut-off value of parenting sense of competence scores has not been determined, this study only discussed the associations between certain variables and parenting sense of competence scores. More research is warranted to determine the optimal cut-off value of parenting sense of competence to accurately identify mothers who are maladjusted to their mother’s role.

## Conclusions

According to our findings, Chinese primiparous women had a relatively good sense of competence and satisfaction in the maternal role, but there is still room for improvement. This study found that parenting sense of competence at 3 months postpartum was associated with primiparous women’s demographic characteristics, family function, and infant characteristics at 1 month postpartum. Health care professionals should pay more attention to primiparous women who are young, with poor economic status, and having an infant in poor health and implement targeted early interventions to improve their parenting sense of competence. In view of the positive effect of good family function on increasing maternal competence in parenting, family members should be encouraged to actively participate in the parenting process and provide high levels of family support. Further work is needed to help primiparous women to initiate breastfeeding and overcome various obstacles and common difficulties.

## Supplementary Information

**Additional file 1: Table S1.** Difference in demographic characteristics for the participants who completed follow-up at 3 months postpartum and those who lost to follow-up.

## Data Availability

The datasets used and/or analysed during the current study are available from the corresponding author on reasonable request.

## Abbreviations

APGAR: Adaptability, Partnership, Growth, Affection, and Resolve
SD: Standard deviation

## References

[1] Mercer RT (2004). Becoming a mother versus maternal role attainment. J Nurs Scholarsh.

[2] Gao LL, Chan SW, Mao Q (2009). Depression, perceived stress, and social support among first-time Chinese mothers and fathers in the postpartum period. Res Nurs Health.

[3] Jansen AJ, Duvekot JJ, Hop WC, Essink-Bot ML, Beckers EA, Karsdorp VH (2007). New insights into fatigue and health-related quality of life after delivery. Acta Obstet Gynecol Scand.

[4] Jones TL, Prinz RJ (2005). Potential roles of parental self-efficacy in parent and child adjustment: a review. Clin Psychol Rev.

[5] Yang XGL, Zhang Z, Wu DH, Zhang Y (2014). The reliability and validity of the Chinese version of parenting sense of competence scale. Chin J Nurs.

[6] Montigny F, Lacharité C (2005). Perceived parental efficacy: concept analysis. J Adv Nurs.

[7] Mohammad KI, Sabbah H, Aldalaykeh M, AL M, ZA K, Creedy D (2021). Informative title: effects of social support, parenting stress and self-efficacy on postpartum depression among adolescent mothers in Jordan. J Clin Nurs.

[8] Macphee D, Miller-Heyl FJ (2010). Ethnic variations in personal social networks and parenting. Child Dev.

[9] Cutrona CE, Troutman BR (1986). Social support, infant temperament, and parenting self-efficacy: a mediational model of postpartum depression. Child Dev.

[10] Ruiz-Zaldibar C, Serrano-Monzó I, Lopez-Dicastillo O, Pumar-Méndez MJ, Iriarte A, Bermejo-Martins E (2021). Parental self-efficacy to promote Children's healthy lifestyles: a pilot and feasibility study. Int J Environ Res Public Health.

[11] Sanders MR, Woolley ML (2005). The relationship between maternal self-efficacy and parenting practices: implications for parent training. Child Care Health Dev.

[12] Albanese AM, Russo GR, Geller PA (2019). The role of parental self-efficacy in parent and child well-being: a systematic review of associated outcomes. Child Care Health Dev.

[13] Amin NAL, Tam WWS, Shorey S (2018). Enhancing first-time parents' self-efficacy: a systematic review and meta analysis of universal parent education interventions' efficacy. Int J Nurs Stud.

[14] Shorey S, Chan SW, Chong YS, He HG (2015). Predictors of maternal parental self-efficacy among Primiparas in the early postnatal period. West J Nurs Res.

[15] Maehara K, Mori E, Tsuchiya M, Iwata H, Sakajo A, Ozawa H (2016). Factors affecting maternal confidence among older and younger Japanese primiparae at one month post-partum. Jpn J Nurs Sci.

[16] Fang Y, Boelens M, Windhorst DA, Raat H, van Grieken A (2021). Factors associated with parenting self-efficacy: a systematic review. J Adv Nurs.

[17] Bryanton J, Gagnon AJ, Hatem M, Johnston C (2008). Predictors of early parenting self-efficacy: results of a prospective cohort study. Nurs Res.

[18] Abuhammad S (2020). Predictors of maternal parenting self-efficacy for infants and toddlers: a Jordanian study. PLoS One.

[19] Yang X, Ke S, Gao LL (2020). Social support, parental role competence and satisfaction among Chinese mothers and fathers in the early postpartum period: a cross-sectional study. Women Birth.

[20] Botha E, Helminen M, Kaunonen M, Lubbe W, Joronen K (2020). Mothers' parenting self-efficacy, satisfaction and perceptions of their infants during the first days postpartum. Midwifery..

[21] Vance AJ, Pan W, Malcolm WH, Brandon DH (2020). Development of parenting self-efficacy in mothers of high-risk infants. Early Hum Dev.

[22] Kiehl EM, White MA (2003). Maternal adaptation during childbearing in Norway, Sweden and the United States. Scand J Caring Sci.

[23] Salonen AH, Kaunonen M, Astedt-Kurki P, Jarvenpaa AL, Isoaho H, Tarkka MT (2009). Parenting self-efficacy after childbirth. J Adv Nurs.

[24] Angley M, Divney A, Magriples U, Kershaw T (2015). Social support, family functioning and parenting competence in adolescent parents. Matern Child Health J.

[25] The Ministry of Public Security of the People's Republic of China (2021). National name report 2021.

[26] State Council of the People's Republic of China (2021). The seventh National Census 2021.

[27] Liu CC, Chen YC, Yeh YP, Hsieh YS (2012). Effects of maternal confidence and competence on maternal parenting stress in newborn care. J Adv Nurs.

[28] Chung FF, Wan GH, Kuo SC, Lin KC, Liu HE (2018). Mother-infant interaction quality and sense of parenting competence at six months postpartum for first-time mothers in Taiwan: a multiple time series design. BMC Pregnancy Childbirth.

[29] Huang MXWC, Huang YS, Yang WF (2016). Correlation between parenting self-efficacy and perceived stress of primiparas. J Guangdong Med Univ..

[30] Hu TY (2019). Effects of exclusive breastfeeding at 6 weeks postpartum on primiparous women general self-efficacy and parenting sense of competence. Electronic journal of practical clinical. Nursing..

[31] Zheng X, Morrell J, Watts K (2018). A quantitative longitudinal study to explore factors which influence maternal self-efficacy among Chinese primiparous women during the initial postpartum period. Midwifery..

[32] Wang Q, Zhang Y, Li X, Ye Z, Huang L, Zhang Y (2021). Exploring maternal self-efficacy of first-time mothers among rural-to-urban floating women: a quantitative longitudinal study in China. Int J Environ Res Public Health.

[33] Law KH, Dimmock J, Guelfi KJ, Nguyen T, Gucciardi D, Jackson B (2019). Stress, depressive symptoms, and maternal self-efficacy in first-time mothers: modelling and predicting change across the first six months of motherhood. Appl Psychol Health Well Being.

[34] Qiu L (2020). A longitudinal study on the intention and behavior of delivery modes among pregnant chinese women using the theory of planned behavior.

[35] WeChat Chinese versions monthly active users over 1.24 billion. 2022. https://baijiahao.baidu.com/s?id=1717127941833187839&wfr=spider&for=pc. Accessed 10 May 2022.

[36] Faul F, Erdfelder E, Buchner A, Lang AG (2009). Statistical power analyses using G*power 3.1: tests for correlation and regression analyses. Behav Res Methods.

[37] Ngai FW, Wai-Chi Chan S, Ip WY (2010). Predictors and correlates of maternal role competence and satisfaction. Nurs Res.

[38] Smilkstein G (1978). The family APGAR: a proposal for a family function test and its use by physicians. J Fam Pract.

[39] Ruan FHDY (2016). Current status of application of the family functioning assessment scale. Chin J Child Health Care.

[40] Gibaud-Wallston J, Wandersman L (1978). Self-esteem and situational stress : factors related to sense of competence in new parents.

[41] Ngai FW, Wai-Chi Chan S, Holroyd E (2007). Translation and validation of a chinese version of the parenting sense of competence scale in chinese mothers. Nurs Res.

[42] Ngai FW, Chan SW (2011). Psychosocial factors and maternal wellbeing: an exploratory path analysis. Int J Nurs Stud.

[43] Shrooti S, Mangala S, Nirmala P, Devkumari S, Dharanidhar B (2016). Perceived maternal role competence among the mothers attending immunization clinics of Dharan. Nepal Int J Community Based Nurs Midwifery.

[44] Esmaelzadeh Saeieh SP, Rahimzadeh MP, Yazdkhasti MP, Torkashvand SM (2017). Perceived social support and maternal competence in Primipara women during pregnancy and after childbirth. Int J Community Based Nurs Midwifery.

[45] Bandura A (1977). Self-efficacy: toward a unifying theory of behavioral change. Psychol Rev.

[46] Glass J, Simon RW, Andersson MA (2016). Parenthood and happiness: effects of work-family reconciliation policies in 22 OECD countries. AJS..

[47] McLeish J, Redshaw M (2017). Mothers' accounts of the impact on emotional wellbeing of organised peer support in pregnancy and early parenthood: a qualitative study. BMC Pregnancy Childbirth..

[48] McLeish J, Redshaw M (2019). “Being the best person that they can be and the best mum”: a qualitative study of community volunteer doula support for disadvantaged mothers before and after birth in England. BMC Pregnancy Childbirth..

[49] Andermo S, Lidin M, Hellenius ML, Nordenfelt A, Nyberg G (2020). “We were all together”- families’ experiences of the health-promoting programme - a healthy generation. BMC Public Health.

[50] Bandura AFWH, Lightsey R. Self-efficacy: the exercise of control. J Cogn Psychother. 1997. 10.1891/0889-8391.13.2.158.

[51] Peñacoba C, Catala P (2019). Associations between breastfeeding and mother-infant relationships: a systematic review. Breastfeed Med.

[52] Hankel MA, Kunseler FC, Oosterman M (2019). Early breastfeeding experiences predict maternal self-efficacy during the transition to parenthood. Breastfeed Med.

[53] Salonen AH, Kaunonen M, Astedt-Kurki P, Jarvenpaa AL, Isoaho H, Tarkka MT (2011). Effectiveness of an internet-based intervention enhancing Finnish parents' parenting satisfaction and parenting self-efficacy during the postpartum period. Midwifery..

[54] de Jager E, Broadbent J, Fuller-Tyszkiewicz M, Skouteris H (2014). The role of psychosocial factors in exclusive breastfeeding to six months postpartum. Midwifery..

