# Supplementary material for: Parenting sense of competence and its predictors among primiparous women: a longitudinal study in China
Source: BMC Pregnancy Childbirth. 2022 Jul 7;22:548. doi: 10.1186/s12884-022-04881-y (PMC9260977; doi:10.1186/s12884-022-04881-y)
Supplement: Supplementary file 1 — Additional file 1: Table S1. Difference in demographic characteristics for the participants who completed follow-up at 3 months postpartum and those who lost to follow-up. [file 12884_2022_4881_MOESM1_ESM.doc]

**Table S1.** Difference in demographic characteristics for the participants who completed follow-up at 3 months postpartum and those who lost to follow-up.

| **Variables** | **Completed（n=743）** | |  | **Uncompleted（n=51）** | | **χ^2^** | ***P*-value** |
| --- | --- | --- | --- | --- | --- | --- | --- |
|  | **n** | **%** |  | **n** | **%** |  |  |
| **Age** |  |  |  |  |  | 0.53 | 0.768 |
| 18-24 | 134 | 18.03 |  | 8 | 15.69 |  |  |
| 25-30 | 516 | 69.45 |  | 35 | 68.63 |  |  |
| ＞30 | 93 | 12.52 |  | 8 | 15.69 |  |  |
| **Education** |  |  |  |  |  | 1.11 | 0.775 |
| Junior high school or below | 61 | 8.21 |  | 6 | 11.76 |  |  |
| Senior high school | 108 | 14.54 |  | 6 | 11.76 |  |  |
| Junior college | 527 | 70.93 |  | 35 | 68.63 |  |  |
| Master degree or higher | 47 | 6.33 |  | 4 | 7.84 |  |  |
| **Employment** |  |  |  |  |  | 0.04 | 0.834 |
| Employed | 477 | 64.20 |  | 32 | 62.75 |  |  |
| Unemployed | 266 | 35.80 |  | 19 | 37.25 |  |  |
| **Whether attended antenatal education** |  |  |  |  |  | 0.39 | 0.534 |
| yes | 595 | 80.08 |  | 39 | 76.47 |  |  |
| no | 148 | 19.92 |  | 12 | 23.53 |  |  |
| **Self-rated sleep quality** |  |  |  |  |  | 0.61 | 0.434 |
| Good | 155 | 20.86 |  | 13 | 25.49 |  |  |
| Not good | 588 | 79.14 |  | 38 | 74.51 |  |  |
| **Self-rated economic status** |  |  |  |  |  | 0.67 | 0.415 |
| Good | 127 | 17.09 |  | 11 | 21.57 |  |  |
| Not good | 616 | 82.91 |  | 40 | 78.43 |  |  |
| **Self-rated health status** |  |  |  |  |  | 0.02 | 0.888 |
| Good | 284 | 38.22 |  | 20 | 39.22 |  |  |
| Not good | 459 | 61.78 |  | 31 | 60.78 |  |  |

**Additional file**

Additional file 1.doc

Table S1. Difference in demographic characteristics for the participants who completed follow-up at 3 months postpartum and those who lost to follow-up. (DOC 56KB)
